# The cost of demand creation activities and voluntary medical male circumcision targeting school-going adolescents in KwaZulu-Natal, South Africa

**DOI:** 10.1371/journal.pone.0179854

**Published:** 2017-06-20

**Authors:** Gavin George, Michael Strauss, Elias Asfaw

**Affiliations:** 1 Health Economics and HIV and AIDS Research Division, University of KwaZulu-Natal, Durban, South Africa; 2 Clinton Health Access Initiative, Health Economics and Financing Analysis Team, Federal Ministry of Health, Addis Ababa, Ethiopia; Cardiff University, UNITED KINGDOM

## Abstract

**Background:**

Voluntary medical male circumcision is an integral part of the South African government’s response to the HIV and AIDS epidemic. However, there remains a limited body of economic analysis on the cost of VMMC programming, and the demand creation activities used to mobilize males, especially among adolescent boys in school. This study addresses this gap by presenting the costs of a VMMC program which adopted two demand creation strategies targeting school-going males in South Africa.

**Methods:**

Cost data was collected from a VMMC program in the KwaZulu-Natal province of South Africa. A retrospective, micro-costing ingredient approach was applied to identify, measure and value resources of two demand creation strategies targeting young males.

**Results:**

The program circumcised 4987 young males between May 2011 and February 2013, at a cost of $127.68 per circumcision. Demand creation activities accounted for 32% of the total cost, HCT contributing 10% with the medical circumcision procedure accounting for 58% of the total cost. Using the first demand creation strategy, 2168 circumcisions were performed at a cost of $149.57 per circumcision. Following this first strategy, a second demand creation strategy was adopted which saw the cost fall to $110.85 per circumcision. More young males were recruited following the second strategy with clinic services more efficiently utilized. Whilst the cost per circumcision of demand activities rose slightly between the first ($39.94) and second ($41.65) strategy, there was a substantial reduction in the cost of the circumcision procedure; $90.01 under the first strategy falling to $60.60 following the adoption of the second demand creation strategy.

**Conclusion:**

Ensuring the optimal use of clinic facilities was the primary driver in reducing the cost per circumcision. This VMMC program has illustrated the value of evaluating progress and instituting changes to attain better cost efficiencies. This adjustment resulted in a substantial reduction in the cost per circumcision.

## Introduction

The results of randomized controlled trials in South Africa, Kenya and Uganda have shown the efficacy of voluntary medical male circumcision (VMMC) in reducing the risk of female to male HIV transmission [1–4]. Following recommendations from the World Health Organization (WHO) in 2007 [5] and models showing the population level impact and cost effectiveness of rapid scale-up [6,7], the South African National Department of Health began implementing a plan in 2010 to circumcise 4.3 million men between the ages 15–49 by 2016 [8]. This fits into the broader global target of reaching 80% coverage in 14 priority countries in eastern and southern Africa (ESA) [6,9]. Significant progress towards this target has been made, with over 2.3 million circumcisions recorded in South Africa by the end of 2015 [10–12]. However, South Africa and other countries in the ESA region are struggling to meet their set targets [12], and it appears unlikely that the 80% coverage target, to maximize population level impact, will be achieved [13].

A significant challenge with scaling up VMMC in South Africa, and one which is common across the region, is the lack of demand for services. While acceptability is fairly high across ESA, studies have shown that reaching 80% of men will require more than simply making services available [14]. In traditionally non-circumcising communities, common barriers to circumcision include costs; fear of pain; threats to masculinity and concerns about adverse events amongst others [15]. Although studies have investigated the barriers and facilitators to VMMC, increasing demand for the procedure remains a challenge with few demand creation programs formally evaluated [16]. While demand creation strategies exist, policy makers and program implementers in many countries, including South Africa, have still struggled to increase VMMC uptake. There have been calls for innovative strategies to increase acceptability and demand for circumcision and frameworks proposed for developing interventions that target demand [13,16,17]. Existing VMMC programs have used different demand creation strategies including community mobilization, mass media and incentivizing target populations through conditional cash transfers, with varying levels of success [18–21].

The focus on scale-up in South Africa’s National Strategic Plan for HIV, TB and STIs 2012–2016, and in the implementation plan for the scale up of VMMC is on increasing the availability of services in healthcare facilities across the country, increasing supply side efficiencies and task shifting; as well as creating demand by increasing information and awareness, social mobilization, and decreasing HIV related stigma. This has been effective to date in reaching early adopters and providing services to those men among whom demand already exists. However, as coverage increases, the demand for VMMC dissipates [22]. Maintaining a steady demand that matches supply is important to increase cost-effectiveness, which has been shown to decrease due to the underutilization of site capacity [23].

An important target group for the HIV response is adolescent males, and there are many HIV prevention interventions targeting the school going population [24]. Studies in the region, including in South Africa, have suggested that targeting school-going adolescents may be cost effective [22, 25]. Research has advocated for the scale up of circumcision among adolescents [25], and in South Africa, increasing VMMC coverage is an important component of the Integrated School Health Policy [26].

In 2014, the costs of providing circumcision services in the general population of men aged 15–49 in South Africa was estimated to be $22,31 per procedure using economic modelling techniques and data collected in 2008 and 2009 [27]. This was substantially lower than the estimated cost for countries in the ESA region which averaged $49.17 per procedure [27], and lower than a modelled estimate of $95.15, using direct and indirect cost estimates from a costing study conducted in Zimbabwe [6]. These estimates of the cost of VMMC are also considerably lower than the cost estimates currently used in the South African Investment Case of $125.50 per circumcision, which were derived from a weighted average of public sector and NGO VMMC program costs in South Africa [24], and $132 per circumcision estimated in a study conducted by the United States President’s Emergency Plan for AIDS Relief and the National Department of Health, and based on facility level costs collected through interviews at 33 different public healthcare facilities around South Africa in 2014 [28].

The gap in the most recently modelled figures and the estimates used in the Investment Case is over $100, which may in part be attributable to the different methods of calculation (a bottom up micro-costing model as opposed to an estimate derived using mixed methods but with a more top down approach and based on past expenditure rather than ingredient cost data), and in part because of the difference in costs when including real world program management and health system costs, as well as the costs associated with creating demand. Cost data in real world settings may also include potential inefficiencies as well as higher level system costs that would not necessarily be included in modelled estimates.

Comparing the costs of demand creation for VMMC is challenging given the different strategies adopted in different settings which often target different populations. There is often a confluence of messaging regarding HIV prevention in general, making it difficult to isolate that which influences demand creation for VMMC specifically. Data are limited, with demand creation activities often conducted discretely as part of service provision, especially within large programs [29]. For many economic evaluations of VMMC service provision, demand creation activities are excluded from the analysis [30].

This paper presents an analysis of the costs of a VMMC program targeting adolescents in South Africa. The program used two different demand creation strategies to mobilize boys for circumcision, and included the provision of HIV counselling and testing (HCT). This study adds to a limited body of economic work on with the cost of VMMC programming, and demand creation activities targeting school-going adolescent males specifically.

## Methods

### Study setting

The cost estimates in this study are based on data collected from an adolescent VMMC program implemented by the Centre for the AIDS Program of Research in South Africa (CAPRISA) in Vulindlela, a rural sub-district in the KwaZulu-Natal province in South Africa. The program included HCT, with VMMC offered to HIV negative boys with the aim of reaching 7761 (70%) of the 11,088 eligible high school boys in the 42 schools targeted. The program ran from mid-2010 to February 2013 and took place in three phases.

The first phase, which started in June 2010 and ran until the end of April 2011 focused on community mobilization, engagement with schools and generation of awareness and provision of information on VMMC [31]. This phase of the program was conducted prior to active recruitment, to garner support for VMMC among the broader community and particularly in schools, as well as gain access to learners in high schools through communication with key role-players such as principals, teachers and governing body members. The costs relating to these initial activities were evenly distributed across the other two phases of the study, in which boys were actively recruited for inclusion in the study.

Phase two took place from May 2011 to February 2012, with active recruitment of boys, using school outreach teams who provided information and offered HCT services in the schools using mobile HCT facilities from Monday to Thursday during term. Recruited boys were then provided transport to and from the CAPRISA clinic in Vulindlela (a fixed site) on Friday afternoons and Saturday mornings to undergo VMMC. In this paper, we refer to this strategy as “Demand Creation Strategy One” (DCS1).

A second, optimized strategy, was adapted in early 2012. In this third phase, which we refer to in this paper as “Demand Creation Strategy Two” (DCS2), boys were recruited by both school outreach teams and peer recruiters from Monday to Friday. Peer recruiters were mostly early adopters of VMMC in the schools recruited by CAPRISA and incentivized to encourage peers to take up VMMC in the program. Recruited boys were then transported to the CAPRISA clinic on Saturdays for both HCT and VMMC. Key characteristics of the two different demand creation strategies are outlined in Table 1.

**Table 1:** Program activities in each demand creation strategy.

Program activities	Demand Creation Strategy 1	Demand Creation Strategy 2
**In school recruitment**	Monday to Thursday	Monday to Friday
**Peer recruiters used**	No	Yes
**Circumcision procedures**	Friday and Saturday	Saturday only
**HCT**	Offered at schools or at the clinic on circumcision days	Conducted pre circumcision at the clinic on a Saturday
**Transport provided**	To and from clinic on circumcision days	To and from clinic on circumcision days
**Education and awareness**	Provided by counsellors in schools as part of Life Orientation curriculum	Provided by counsellors in schools as part of Life Orientation curriculum
**Post-operative assessments**	In schools the following week	In schools the following week

### Costing perspective

A retrospective, micro-costing approach, was applied to identify and value resources of two demand creation strategies targeting adolescents to increase uptake of VMMC services. Following micro-costing principles, steps included framing the cost analysis, developing the cost inventory, quantifying cost and calculating summary measures [32]. To substantiate the costing evidence from the sites, costing data were collected at the CAPRISA head office with the help of program and finance staff. All relevant resource inputs including labor, supplies, capital items/equipment, infrastructure costs, transportation costs and program management and supervision costs were collected and analyzed. The costing was done from the health care provider perspective considering both the variable and fixed resource inputs. A detailed costing sheet and structured interview guide were prepared to retrieve the resource ingredients and respective unit cost data. In addition, service utilization data were retrieved from program monitoring and evaluation reports.

**Fig 1:**
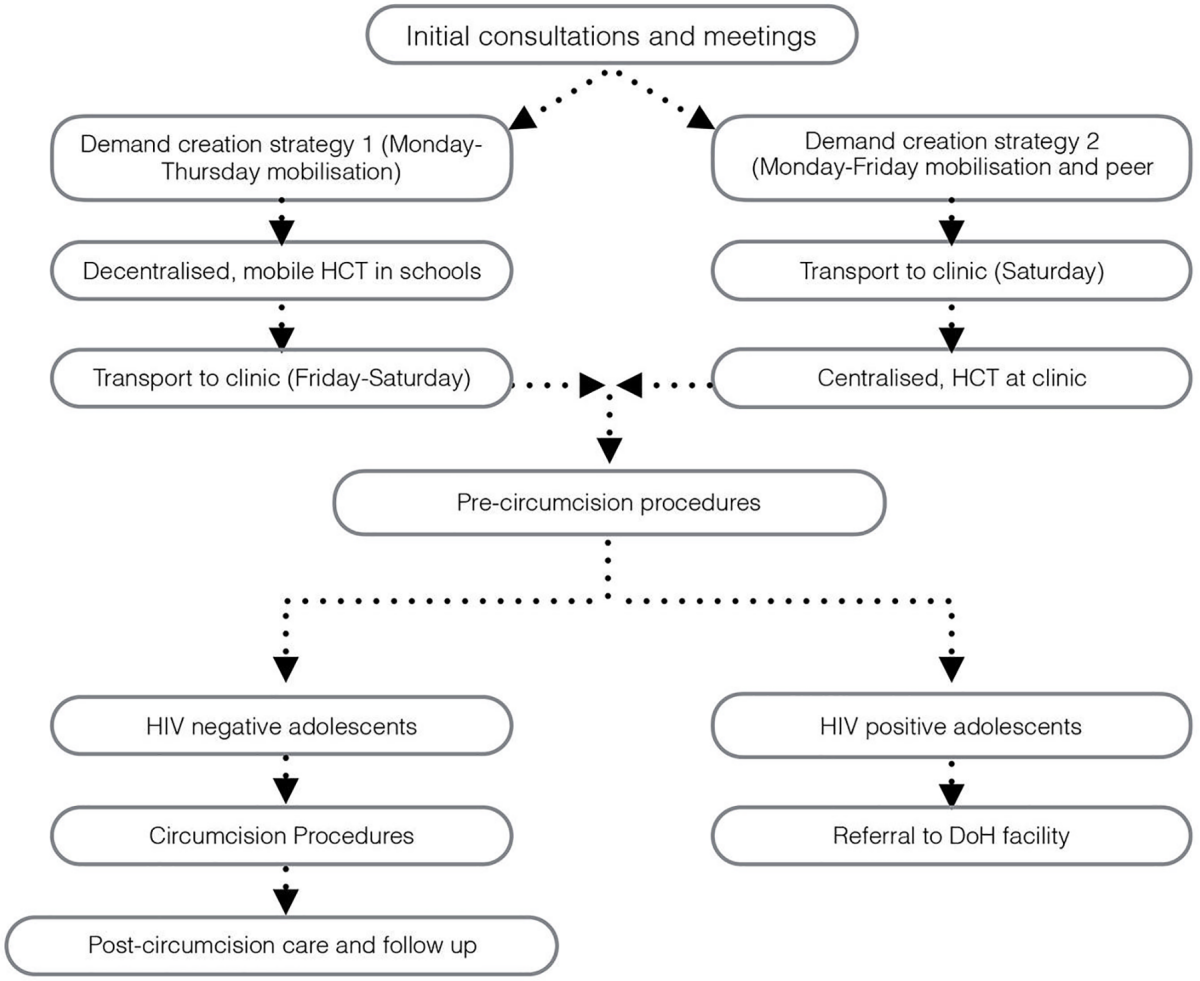
Costing map for the adolescent targeted VMMC services.

As suggested in Drummond et al. [33], the value of each resource ingredient was estimated and calculated with its corresponding unit price. A costing map was developed explicitly to identify the relevant resource items across the adolescent targeted VMMC intervention (See Fig 1).

### Costing categories

Costs were placed in three categories for analysis. The first categorization was according to thematic intervention areas, which we define as: HCT activities; VMMC activities and demand creation activities. HCT activities included pre- and post-test counselling and the HIV test. VMMC activities included counselling, the procedure and post-surgery care. Demand creation activities included initial consultations and information sessions, adolescent mobilization and peer recruitment, and the transportation of boys to the clinic.

The second categorization was according to intervention activities, which reflects the flow of the VMMC program activities. These activities included: initial consultations (with community members and school management); adolescent mobilization (school outreach and mobilization); transportation services (for the boys to and from the clinic on circumcision days); pre-circumcision procedures (including HCT, pre-circumcision care); costs for HIV positive boys (not eligible for inclusion, but who engaged in a “mock” procedure to protect their HIV status); post-surgery care (at the clinic including counselling, lunch and entertainment); follow-up assessment at schools (on the Monday following the procedure); and management and supervision (including overall program administration). Details documenting the program process can be found elsewhere [31].

Table 2 gives a description of the activities in the program and resources used.

**Table 2:** Description of activities and resource inputs required for each activity.

Activity	Description
Initial consultations and meetings	A small group, comprising the HCT program manager and one or two counsellors went into communities, presenting information and seeking support at community forums or school management meetings. On some occasions, a tent was set up to facilitate meetings, with light refreshments and seating provided. Unit costs were collected from accounting and human resource records at CAPRISA. Counsellor wages were commensurate with the KwaZulu-Natal Department of Health counsellors. Costs of equipment and refreshments were estimated from expenditure reports, and the cost of the Toyota Hilux, used for transport, was estimated using an annuitized cost based on purchase price and running costs as well as the prevailing interest rate in South Africa. These costs were apportioned between DCS1 and DCS2 per the duration of each strategy.
Adolescent mobilization	Four counsellors, accompanied by a driver, provided VMMC information to learners at schools. The team recruited learners for VMMC, and under DCS1, provided HCT in schools. Under DCS2, peer recruiters were also used to mobilise boys for VMMC. In most cases, adolescent mobilization activities were integrated into existing life orientation programs and classes, but in some instances, adolescent mobilization took place during student recess or after school. Staff salary information was collected from CAPRISA. Although CAPRISA salary information is confidential, hourly wage rates are commensurate with healthcare workers in the public sector. Peer recruiters were reimbursed with cell phone airtime, and small prizes handed out at functions that were held periodically for the boys involved–there was no explicit payment for involvement as a peer recruiter. Teams utilized a Toyota Hilux belonging to CAPRISA to access schools.
Transportation	Learners were provided with transport from their community (usually their school, a local community hall or other central location) to and from the clinic. Services were provided by local minibus drivers for a set fee, dependant on distance from the clinic and pick-up points, and number of trips undertaken.
Pre-circumcision procedures	Services included HCT, screening for sexually transmitted infections, and general health eligibility checks for VMMC. Under DCS1, HCT and STI screening was conducted in schools during the week using a mobile testing vehicle or venues provided on the school premises, with eligibility checks conducted at the clinic on VMMC days. Under DCS2, these activities all took place at the clinic prior to the procedure. At the clinic, three clinical rooms were hired; two days a week under DCS1, and one day a week under DCS2.
HIV positive males	HIV positive males were not eligible for circumcision, but to protect their HIV status, they underwent a mock procedure (DCS2 only), in which they received attention from a nurse in one of the circumcision rooms to reduce the potential stigma and discrimination. These boys were referred to CAPRISA’s HIV treatment program, and to the department of health clinics if they still wanted to undergo circumcision. Resource use for HIV positive boys was limited to the space used and time spent by healthcare workers in conducting the mock procedure.
Circumcision procedures	HIV negative boys that passed all health eligibility checks underwent the circumcision procedure. Two sessional nurses supported a sessional doctor in conducting the procedure using the forceps guided surgical method. Sterilised, disposable circumcision kits were used for the procedure, under nurse administered local anesthetic. The cost of the circumcision kits used was subsidised by donor partners, so for this study, an estimate based of $23 per kit was used, with sensitivity analysis conducted to reflect the decline in the price of these kits in South Africa to $15.10 in more recent studies [27]. Clinical rooms were hired for circumcision days, and were fully equipped to accommodate the procedure. Thus, the capital items that were needed to conduct VMMC are included in these costs. Doctors and nurses were hired only on circumcision days, and were paid an hourly rate in line with current sessional rates for healthcare workers in South Africa.
Post-surgery care	After undergoing circumcision and waiting to be transported home, boys were provided with a room to recover in. Light refreshments were provided (sandwiches, crisps and sodas) and various games were provided to keep them entertained. Boys were attended to by healthcare workers when required and supervised by a site administrator until all procedures had been completed, at which point they were taken back to their community.
Follow-up assessment	On the Monday following VMMC, a team of three nurses accompanied by a driver were responsible for conducting follow-up assessments with the boys that had undergone procedures to monitor and treat potential adverse events.
Management and supervision	Along with the teams responsible for adolescent mobilization, HCT and VMMC, a project manager and administrator oversaw logistics, and ongoing management of relationships with communities and schools. The costs of these inputs were apportioned equally between demand creation, HCT and VMMC activities; and between DCS1 and DCS2 per the duration of each strategy.

The third categorization, used economic cost categories (or ingredients) for comparability with other costing studies [23,27,34]. These categories included labor, capital (equipment), infrastructure, and consumables. Additionally, due to the design of the program, transportation costs were separated out, and a category for cross-cutting costs was included, covering program management, supervision, administration and associated costs.

The service inputs were valued in South African Rand (ZAR) at the base year (March 2012-Feb 2013) and converted to USD dollars using the mid-point exchange rate during that period [35]. All capital item costs were annuitized and discounted at 3%, and adjusted for inflation at prevailing rates during that period [36,33]. The adjusted inflation conversion factor is taken from the International Monetary Fund (World Economic Outlook Database for South Africa– 10 years from 2005 to 2014) [37].

The final comparisons are conducted using the average unit cost summary measure to highlight the cost efficiency and cost savings for the different demand creation strategies. The final unit cost summary measures are calculated for the three key VMMC related activities, that is average cost per demand creation activity only, average cost per HCT service only and average cost per VMMC service only. Microsoft excel 2013 was used for the data analysis.

### Ethics

Full ethical approval for this study was obtained from the Biomedical Research Ethics Committee at the University of KwaZulu-Natal (Ref: BF128/11) and the study followed all standard ethics procedures for the collection and analysis of data.

## Results

### Cost by thematic intervention

Table 3 illustrates the costs of the program by thematic area, first for the entire program including DCS1 and DCS2, and then separately for each of the demand creation strategies. Overall, the average cost, for the entire program (including both demand creation strategies), for all 4987 circumcisions conducted between May 2011 and February 2013 was $127.68 per circumcision. Demand creation activities accounted for 32% of the total cost with the HCT component contributing 10% and the medical circumcision procedure accounting for 58% of the total cost. In DCS1, 2168 circumcisions were performed between May 2011 and February 2012 –an average of 217 procedures per month–at a cost of $149.57 per circumcision. From March 2012 to February 2013, in DCS2, this cost fell to $110.85 per circumcision, while at the same time the average number of circumcisions increased to 235 per month. It is important to note that the number of days the circumcision facility was operational fell from two per week in DCS1 to only once a week in DCS2, meaning that the average number of circumcisions per day more than doubled during DCS2, resulting in a lower cost per procedure.

**Table 3:** Costs by thematic intervention activity.

Number of Circumcisions	Total (4987)			Demand Creation Strategy 1 (2168)		Demand Creation Strategy 2 (2891)	
Thematic intervention activity	Cost, 2013 USD	Cost per circumcision, 2013 USD	Share of total costs	Cost per circumcision, 2013 USD	Share of total costs	Cost per circumcision, 2013 USD	Share of total costs
Demand creation activities	$204 013.51	$40.91	32.04%	$39.94	26.70%	$41.65	37.58%
HIV counseling & testing	$66 580.89	$13.35	10.46%	$19.53	13.06%	$8.60	7.76%
VMMC service	$366 154.20	$73.42	57.50%	$90.09	60.24%	$60.60	54.67%
**Total costs**	**$636 748.59**	**$127.68**	**100%**	**$149.57**	**100%**	**$110.85**	**100%**

In DCS1, the number of HIV tests performed per circumcision was 1.34. This was partly due to inefficiencies linked to boys signing up for VMMC and testing during the week at the mobile testing facilities at schools, but not presenting at the clinic on the Friday or Saturday when the procedure was due to be performed. This ratio fell to 1.01 tests per circumcision under DCS2, reflecting the increased efficiency of the strategy–to test boys for HIV on the same day as the circumcision procedures–and partly accounts for the decrease in the cost of HCT per boy circumcised from $19.53 in DCS1 to $8.60 in DCS2.

In DCS2, costs were redirected from VMMC service delivery to demand creation activities, resulting in the costs of the VMMC component of the programme falling by a third from $90.09 to $60.60. The analysis reveals that demand creation costs under DCS1 constituted just 27% of the program costs per circumcision, as opposed to the demand creation costs under DCS2 which accounted for 38% of the cost of the program per circumcision performed. This is partly a reflection of the fact that demand creation activities under DCS2 were conducted over five days a week rather than four days a week under DCS1, and partly a reflection of the overall lower cost of the program under DCS2.

### Cost by intervention activity

Table 4 shows the breakdown of costs per activity within the CAPRISA VMMC program. The costs directly associated with adolescent mobilization and recruitment of boys in schools went up considerably from $14.54 to $20.40 per circumcision performed, accounting for approximately one tenth of the cost in DCS1, but almost one fifth of the cost in DCS2. This partially reflects the increased number of days dedicated to adolescent mobilization in DCS2, and the fact that fieldworkers were not focused on both adolescent mobilization and HIV testing activities in the schools as was the case in DCS1. Transportation for the boys to get to the clinic fell slightly on average per circumcision from $12.68 in DCS1 to $10.65 in DCS 2, because of the utilization of transportation on one day rather than two in DCS 1.

**Table 4:** Costs per intervention activity.

	Demand Creation Strategy 1 and 2			Demand Creation Strategy 1 only		Demand Creation Strategy 2 only	
Intervention Activities	Cost, 2013 USD	Cost per circumcision, 2013 USD	Share of total costs	Cost per circumcision, 2013 USD	Share of total costs	Cost per circumcision, 2013 USD	Share of total costs
Initial consultation and meetings	$31 956.39	$6.41	5.02%	$7.37	4.93%	$5.67	5.11%
Adolescent mobilization activities	$89 043.05	$1786	13.98%	$14.54	9.72%	$20.40	18.41%
Transportation services	$57 502.18	$11.53	9.03%	$12.68	8.48%	$10.65	9.60%
Pre-circumcision procedures (HCT, STI counseling and screening)	$40 321.77	$8.09	6.33%	$13.97	9.34%	$3.56	3.21%
HIV positive tested adolescent youths	$747.23	$0.15	0.12%	$0.21	0.14%	$0.10	0.09%
Circumcision procedures	$246 331.71	$49.39	38.69%	$60.56	40.49%	$40.81	36.81%
Post-surgery (circumcision) care	$34 126.25	$6.84	5.36%	$10.01	6.70%	$4.40	3.97%
Follow-up assessments	$60 184.34	$12.07	9.45%	$14.17	9.47%	$10.45	9.43%
Management and supervision costs	$76 535.67	$15.35	12.02%	$16.05	10.73%	$14.81	13.36%
**Total costs**	**$636 748.59**	**$127.68**	**100%**	**$149.57**	**100%**	**$110.85**	**100%**

The costs of pre-circumcision activities, which included HCT and screening for STIs, decreased from $13.97 to $3.56 per circumcision by offering these services at the clinic on the Saturday prior to the procedure in DCS2, rather than at schools throughout the week under DCS1. The cost directly associated with the circumcision procedure accounted for about 40% of the total cost under both strategies. However, the unit cost per individual tested fell by almost $20 from $60.56 in DCS1 to $40.81 in DCS2. This is largely because of the decrease in the cost of the staff used to conduct procedures in DCS2, and more optimal utilization of the clinic. The cost of post-surgery care and follow up assessments, which accounted for 5.36% and 9.45% of the total costs of the program overall respectively, also fell–from $10.01 in DCS1 to $4.40 in DCS2 and from $14.17 in DCS1 to $10.45 in DCS2 respectively–largely due to the increased efficiency of site capacity utilization.

### Cost by economic resource category

Table 5 reveals the cost by economic ingredient, to allow for comparison with previous studies. In terms of cost drivers, labor was found to be the most substantial component, comprising 51% of the cost of the program overall. This partly reflects the high usage of labor for adolescent mobilization activities, recruitment and undertaking the procedure; but also that staff involved in outreach activities were often required to travel far in order to reach some of the schools included in the sample. Labor costs were reduced substantially, from $80.48 in DCS1 to $52.90 in DCS2. In DCS1, doctors and nurses (earning a higher wage) were used for two days a week to conduct procedures, with counsellors (earning a lower wage) conducting adolescent mobilization activities on four days a week. In DCS2, the emphasis on adolescent mobilization increased with counsellors recruiting for five days a week, and nurses and doctors conducting VMMC procedures on one day.

**Table 5:** Costs per economic costing ingredient.

	Demand Creation Strategy 1 and 2			Demand Creation Strategy 1		Demand Creation Strategy 2	
Costing ingredient	Cost, 2013 USD	Cost per circumcision, 2013 USD	Share of total costs	Cost per circumcision, 2013 USD	Share of total costs	Cost per circumcision, 2013 USD	Share of total costs
Labor costs	$323 604.13	$64.89	50.82%	$80.48	53.81%	$52.90	47.72%
Consumables	$125 099.55	$25.09	19.65%	$25.90	17.32%	$24.46	22.06%
Capital items (Equipment)	$11 904.10	$2.39	1.87%	$2.50	1.67%	$2.30	2.08%
Transportation cost	$78 608.81	$15.76	12.35%	$17.86	11.94%	$14.15	12.76%
Infrastructure	$20 039.68	$4.02	3.15%	$6.78	4.53%	$1.89	1.71%
Cross-cutting costs	$77 492.32	$15.54	12.17%	$16.05	1073%	$15.15	13.67%
**Total costs**	**$636 748.59**	**$127.68**	**100%**	**$149.57**	**100%**	**$110.85**	**100%**

The cost of consumables ($25.09), accounted for almost 20% of the total cost of the program. A large portion of the consumables cost per boy was attributed to the cost of the disposable VMMC kit ($23), which contained the consumables used to perform the procedures. In the sensitivity analysis, which used current disposable VMMC kit prices in South Africa of $15.10 [28] as an estimate of cost, the cost of consumables was considerably lower (a cost of $18 in DCS1 and $16.56 in DCS2) and leading to a substantially lower total cost per procedure ($141.67 in DCS1 and $102.95 in DCS2). Capital items accounted for a relatively small proportion of the costs in this analysis, as most of the equipment needed for conducting procedures was included in the facility rental cost, and thus included under infrastructure. The costs of infrastructure and equipment were minimal, as the space used was rented on a daily basis for two days a week in DCS1, and for one day a week in DCS2. This also resulted in the cost associated with infrastructure being substantially lower in DCS2 than in DCS1.

The transport ingredient is treated as a separate economic resource category, as it constitutes a substantial portion of the total cost in both demand creation strategies. The average cost for transport per boy circumcised decreased to $14.15 per circumcision in DCS2, from $17.86 per circumcision in DCS1. Cross-cutting costs (see Table 2) also decreased slightly from $16.05 per circumcision in DCS1 to $15.15 in DCS2.

## Discussion

VMMC has been an important prevention tool in the South African NSP 2012–2016 and will undoubtedly remain so in the next iteration, and will continue to form part of regional strategies. Whilst accessing school-going males may prove easier than adult males, creating demand for VMMC remains challenging even amongst school-going adolescents. With global targets for VMMC set at 80% of males, significant work is required to identify the demand creation strategies required to realize this figure. CAPRISA set out to circumcise 7761 boys within their catchment population and, despite their demand creation efforts, fell short of their more modest 70% target.

There remains however, a dearth of available literature evaluating VMMC demand creation strategies targeting school-going males and specifically, any undertaking economic analysis.

This study addresses this gap by describing and costing CAPRISA’s integrated HIV testing and VMMC program targeting school-going males. The study costed two DCS’s adopted by CAPRISA with the latter being implemented at a lower unit cost than the initial DCS: $110.85 vs $149.57. These figures are comparable to other South African VMMC cost estimates of $125.50 [24] and $132 [28]. Importantly, this study distinguishes costs associated with creating demand for VMMC from those costs associated with the actual procedure.

One of the major challenges inhibiting scale up of VMMC is the cost born by potential clients in accessing services [15]. A significant component of the DCS’s adopted by CAPRISA was to remove this cost by providing transport to and from the clinic. In addition, the successful recruitment of young males was determined by several factors, including provision of information at schools by counsellors and subsequent recruitment. DCS2 utilized peer recruiters in addition to a roaming team of counsellors. It is difficult to determine the effectiveness of these activities given the absence of a counterfactual. However, as was evident in DCS2, sufficient numbers of young males were recruited to ensure a more optimal utilization of clinic facilities and staff.

Ensuring the optimal utilization of services will improve the cost-effectiveness of VMMC programs [25]. Within DCS1, CAPRISA was unable to recruit enough young males to ensure the clinic services were optimally used over two days. Undertaking the procedure on only one day (in DCS2) allowed for additional recruitment time and subsequent optimal use of clinic services, leading to a substantial cost saving from DCS1 to DCS2. Staffing costs remain a large part of the cost of VMMC programs [13,24,27,28] and this study affirms these findings by illustrating the difference in the cost of the VMMC procedure from DCS1 ($90.09 per circumcision) to DCS2 ($60.60). Task-shifting, with trained nurses undertaking the procedure, possibly by using non-surgical methods, could potentially reduce this this cost even further [38].

The shift from offering HCT at schools prior to enrolment to providing HCT at the clinics prior to undertaking the procedure substantially reduced the average cost per procedure. These costs decreased by more than half from DCS1 ($19.53) to DCS2 ($8.60). The major contributing factor behind the decrease in costs was the improved test to circumcision ratio, also reflecting the efficiency gains from site use capacity i.e. having all the boys that undergo VMMC tested on the day of the procedure, rather than the use of the decentralized mobile testing facility, where fewer boys per day were tested. Far more young males were undergoing HCT and getting circumcised under DCS2 compared to DCS1, which also helped to optimize utilization of the transportation services used by the program. Importantly, CAPRISA addressed the potential stigma of a positive result at the clinic by ensuring the young males underwent the mock circumcision procedure.

### Limitations

The cost of adverse events was not factored in to this study. Being a retrospective analysis, and given that adverse events would have been dealt with outside of this specific program, cost data on adverse events were difficult to obtain. However, based on the low number and severity of adverse events in the program (1.2% and mostly minor and self-resolving) [31], as well as findings from previous work indicating minor costs associated with adverse events [39], we estimate that these costs would be a small proportion of the total cost of the program.

The methods adopted here are also subject to two primary limitations. Firstly, given that the study is a retrospective analysis, expenditure reports and accounting records were used to estimate the costs of some of the resources used and in some cases, gross costing techniques were used to estimate unit costs. This mixture of methods may lead to slight over- or under-estimates of true costs. Secondly, the cost estimates used in this study pertain to a program implemented in the context of a research study. Thus, the costs when rolled out in public settings may vary, especially when taking into consideration higher level system costs that would be involved in ensuring a well-coordinated response that would be required if the adaptive demand creation strategy costed here were to be scaled up.

## Conclusion

VMMC programs and demand creation strategies in particular need to be adaptable to context, and need to have the flexibility to adjust to the setting and the target population. In this program, CAPRISA adapted their strategy after one year and increased demand creation activities, resulting in the recruitment of a larger numbers of boys and the optimal utilization of the clinic. The flexibility of the program allowed for the reallocation of resources to affect these changes, which ultimately led to an improved cost to circumcision ratio. This VMMC program has illustrated the value of evaluating progress and instituting changes to attain cost efficiencies.
